# Winter feeding during the spawning migration of capelin north of Iceland

**DOI:** 10.1111/jfb.70677

**Published:** 2026-09-30

**Authors:** Hildur Petursdottir, Teresa Silva, Sigurdur Th. Jonsson, Warsha Singh

**Affiliations:** ^1^ Marine and Freshwater Research Institute Hafnarfjordur Iceland

**Keywords:** capelin, diel feeding, Iceland, spawning migration, stomach contents, winter feeding

## Abstract

Winter feeding during the spawning migration of capelin is often assumed to be minimal, because energy is allocated to reproduction. However, January–February surveys north of Iceland in 2018 and 2019 showed that prey occurred in 64% and 62% of 423 stomachs examined, respectively. Copepods (*Calanus* spp.) and euphausiids dominated the diet, and mean feeding intensity tended to be higher by day than by night. Mean winter feeding intensity was lower than autumn values estimated using the same approach, but feeding was still present. These observations indicate that capelin feed during the winter spawning migration.

Capelin, *Mallotus villosus* (Müller, 1776), is a key mid‐trophic forage fish in Arctic and sub‐Arctic ecosystems, transferring energy from zooplankton to a wide range of predators (Astthorsson & Gislason, 1997; Pálsson & Björnsson, 2011; Víkingsson et al., 2015; Vilhjalmsson, 1994). In the Iceland‐East Greenland‐Jan Mayen (IEGJM) region, feeding is typically intense in summer–autumn, whereas feeding during the winter spawning migration is commonly regarded as reduced (Astthorsson & Gislason, 1997; Petursdottir et al., 2026; Sigurdsson, 1992; Sigurdsson & Astthorsson, 1991). This assumption is also supported by evidence that adult capelin build lipid reserves during summer feeding and largely use these reserves for gonad development and fuelling the spawning migration (Vilhjalmsson, 2002).

Historical stomach‐content studies north of Iceland reported a high prevalence of empty stomachs in late autumn and early winter, with the proportion of empty stomachs increasing into December (Astthorsson & Gislason, 1997; Sigurdsson, 1992; Sigurdsson & Astthorsson, 1991). Meanwhile, capelin distribution and migration timing have shifted since the early 2000s (Pálsson et al., 2012; Singh et al., 2020; Singh et al., 2025; Vilhjalmsson, 2007). These changes include shifts westward in the spatial extent of feeding areas and in the timing of arrival to the shelf north of Iceland prior to the spawning migration. Such shifts can alter when and where migrating fish encounter prey, making updated winter observations ecologically relevant.

Here we report a concise field‐based observation from spawning‐migration surveys conducted along the Icelandic continental shelf north of Iceland in January–February 2018 and 2019. In total, 330 fish were sampled from 36 trawls in 2018 and 93 fish from 10 trawls in 2019. Our objective was to quantify winter feeding intensity (Total Fullness Index, TFI and Partial Fullness Index, PFI) and compare it with autumn values estimated using the same approach (Petursdottir et al., 2026). We also quantified prey occurrence, described dominant prey taxa by size class and tested whether feeding intensity differed between day and night under winter light conditions. Because sampling month, area and protocols differ among historical records from the early 1990s, we use earlier work only for comparison rather than a formal time trend.

Capelin were sampled during two spawning‐migration surveys conducted by the Marine and Freshwater Research Institute north of Iceland (18–30 January 2018 and 25 January–23 February 2019) using targeted pelagic trawl stations selected from acoustic backscatter where high densities were observed. At each station, stomachs from 10 randomly selected fish were removed and frozen immediately. In total, stomach contents were analysed from 330 individuals in 2018 and 93 in 2019 (*n* = 423; 11.5–19.5 cm total length). Stomach contents were processed using the same laboratory protocol and fullness indices (PFI and TFI) as those used in the autumn study (Petursdottir et al., 2026), allowing direct comparison of feeding intensity between seasons. Prey were identified to the lowest feasible taxonomic level, oven‐dried (60–70°C for ≥24 h) and weighed. Feeding intensity was quantified using the PFI (Lilly & Fleming, 1981), which standardizes prey dry mass by fish length to allow comparisons across size classes and is given as:

(1) $${\mathrm{PFI}}_{i,k}=\frac{S_{i,k}}{L_{k}^{3}}\;\times \;10^{4}$$

where *S*_*i,k*_ is the dry weight (g) of prey species *i* found in the stomach of predator *k*, and L_*k*_ is the total length (cm) of predator *k*.

TFI was calculated as the sum of PFI across prey groups. For statistical analyses, each trawl was treated as the independent sampling unit, and individual‐fish TFI/PFI values were summarized within each trawl before further analysis. For spatial presentation, PFI/TFI values were averaged across the sampled fish within each trawl, usually 10 fish per trawl (Figure 1). Fish were grouped into <14 cm and ≥14 cm total length (Table 1), which is a practical threshold associated with maturity (Jourdain et al., 2021).

**FIGURE 1:**
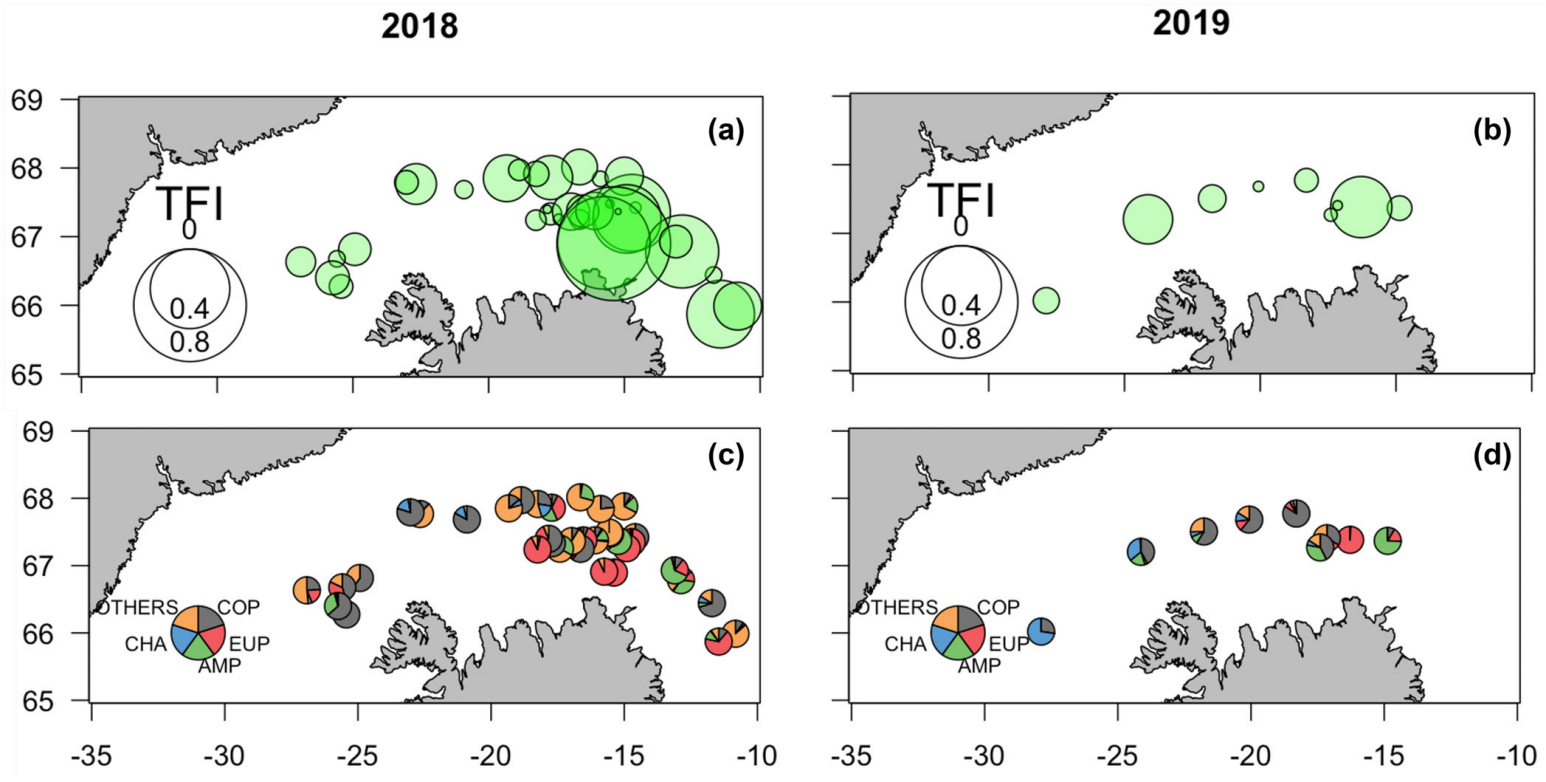
Total Fullness Index (TFI; panels a, b) and Partial Fullness Index (PFI; panels c, d) for capelin across sampling locations during winter surveys north of Iceland in 2018 (a, c) and 2019 (b, d). COP = copepods, EUP = euphausiids, AMP = amphipods, CHA = chaetognaths, OTHERS = all remaining prey groups.

**TABLE 1:** Dominant prey taxa within major prey groups in the diets of small (<14 cm) and large (≥14 cm) capelin sampled north of Iceland during winter 2018 and 2019.

Taxon year	Capelin <14 cm		Capelin ≥14 cm	
	2018	2019	2018	2019
No. stomachs analysed	75	13	255	80
No. empty stomachs	22	9	96	26
Copepoda				
*Calanus finmarchicus*	50	53	28	37
*C. glacialis*			2	4
*C. hyperboreus*	1		9	28
*Calanus* spp.	45	39	47	26
*Euchaeta* spp.	1		5	4
*Metridia longa*	3	6	9	1
*Pseudocalanus* spp.		1		
Euphausiacea				
Euphausiidae		100	36	86
*Meganyctiphanes norvegica*			29	
*Thysanoessa inermis*			41	
*T. longicaudata*				14
Amphipoda				
*Themisto abyssorum*			35	2
*T. libellula*				40
*Themisto* spp.			65	58
Chaetognatha	100	100	100	100
Other				
Capelin larvae			56	53
*Limacina retroversa*			43	47
Hydrozoa			1	

*Note*: Values represent the percentage contribution of each taxon within its prey group, year and size class; values within each prey group sum to 100%. Rare taxa are omitted for brevity. The number of stomachs analysed and the number of empty stomachs are shown for each year and size class.

Capelin are known to exhibit diel vertical migration (Mowbray, 2002; Silva et al., 2025). To assess diel variation in feeding, samples were classified as daytime or nighttime based on each station's date, location and sampling time, using solar elevation (sun angle) as the reference (Thieurmel & Elmarhraoui, 2022). Differences in TFI between day and night were tested separately for each winter using Welch's *t*‐test. One trawl in 2019 could not be classified as day or night and was excluded from the diel comparison.

Stomachs containing prey were common in both winters, occurring in 64.2% of fish in 2018 and 62.4% in 2019 (Table 1), while mean trawl‐level TFI was 0.050 ± 0.021 SE in 2018 and 0.065 ± 0.027 SE in 2019, with spatial variability among trawls (Figure 1). Diets were dominated by copepods and euphausiids in both years (Figure 1). Among copepods, *Calanus finmarchicus* and *Calanus* spp. made up most of the copepods, whereas euphausiids were mainly *Thysanoessa inermis* and *Meganyctiphanes norvegica* (Table 1). Amphipods and chaetognaths contributed noticeably and were more evident in larger fish. The ‘other’ prey category included capelin larvae and the pteropod *Limacina retroversa* (Table 1). As in autumn (Petursdottir et al., 2026), copepods and euphausiids dominated the diet, whereas amphipods and chaetognaths appeared more prominent in winter.

Mean TFI was higher during daytime than nighttime trawls in both winters, although the differences were not statistically significant (2018: day 0.092 ± 0.078 SE, *n* = 9 trawls; night 0.036 ± 0.012 SE, *n* = 27 trawls; Welch's *t*‐test, *p* = 0.502; 2019: day 0.107 ± 0.079 SE, *n* = 3 trawls; night 0.031 ± 0.014 SE, *n* = 6 trawls; Welch's *t*‐test, *p* = 0.439). This pattern is consistent with capelin relying strongly on vision when feeding, with winter light conditions potentially limiting effective foraging to the short daylight period (Hassel et al., 1991). Daylight duration during the winter surveys was approximately 4–9 h day^−1^, compared with approximately 9–14 h day^−1^ during the autumn surveys, based on sunrise and sunset times calculated for the sampling date and position using the suncalc package (Thieurmel & Elmarhraoui, 2022). For context, autumn data did not show a similarly consistent day‐night pattern (Petursdottir et al., 2026), suggesting diel effects may be more pronounced under winter light conditions.

A directly comparable seasonal contrast based on trawl‐level values showed lower mean TFI in winter 2019 than in autumn 2018, although the difference was not statistically significant (Welch's *t*‐test, *p* = 0.468). Mean TFI decreased from 0.097 ± 0.034 SE (*n* = 35 trawls) in autumn 2018 to 0.065 ± 0.027 SE (*n* = 10 trawls) in winter 2019, indicating reduced but still evident feeding during migration. We did not use winter 2018 because it occurred before the autumn 2018 sampling period. Winter 2019 therefore provides the more appropriate comparison with autumn 2018.

For comparison, Dalpadado and Mowbray (2013) reported mean TFI values of 0.036–0.100 off Newfoundland in May–June and 0.129–0.168 in the Barents Sea in August–September. Although these values come from different regions, seasons and populations, they provide a broad point of reference suggesting our winter TFI falls within the lower end of TFI values reported for feeding capelin.

These observations demonstrate that capelin can feed frequently in winter during the spawning migration in some years, contradicting a simple expectation of uniformly ‘empty’ stomachs in winter in historical studies (Astthorsson & Gislason, 1997; Sigurdsson, 1992; Sigurdsson & Astthorsson, 1991). Continued monitoring, together with prey‐field sampling and measurements of fish condition, would help determine how often this occurs and its energetic significance. Interannual variation in winter feeding could influence capelin condition and overwinter survival, particularly if reduced feeding increases reliance on stored energy reserves. It may also affect exposure to predators and the contribution of capelin to predator diets during winter, although these links were not evaluated here.

In summary, capelin sampled during spawning‐migration surveys north of Iceland in 2018 and 2019 often contained prey. Diets were dominated by copepods and euphausiids, and mean feeding intensity tended to be higher during the day than at night. A directly comparable autumn–winter contrast showed lower mean feeding intensity in winter than in autumn, although feeding remained evident during winter, indicating that winter feeding during the spawning migrations should be considered when interpreting winter energy budgets and food‐web dynamics in subarctic shelf ecosystems.

## AUTHOR CONTRIBUTIONS

H.P. conceptualized the study and wrote the original draft. T.S. prepared the figure. All co‐authors reviewed and edited the manuscript and approved the final version.

## Data Availability

The data that support the findings of this study are available on request from the corresponding author. The data are not publicly available due to privacy or ethical restrictions.
